# Hypothesized pharmacogenomic and medication influences on tetrahydrocannabinol and cannabidiol metabolism in a cohort of unselected oral cannabis users

**DOI:** 10.1186/s42238-024-00256-6

**Published:** 2025-01-04

**Authors:** Jessica A. Wright, Linda Huang, Basant E. Katamesh, Siddhant Yadav, Abhinav Singla, Ann Vincent

**Affiliations:** 1 Pharmacy Services, Mayo Clinic College of Medicine and Science, 200 First St SW, Rochester, MN 55905 USA; 2 Division of General Internal Medicine, Mayo Clinic College of Medicine and Science, 200 First St SW, Rochester, MN 55905 USA

**Keywords:** Pharmacogenomics, Potential drug-gene interactions, CBD, THC, CYP450 enzyme, Pharmacology, Cannabinoid levels

## Abstract

**Background:**

Differences in cannabinoid metabolism and patient responses can arise even with equivalent doses and formulations. Genetic polymorphisms in genes responsible for cannabinoid metabolism and medications that alter CYP450 pathways responsible for metabolism of cannabinoids may account for some of this variability.

**Materials and methods:**

A retrospective chart review was conducted on a cohort of unselected patients who had previously completed pharmacogenomic testing and reported oral cannabis use, as defined as “oral” or “by mouth” route of administration. The objective was to identify atypical variants and medications in this cohort and formulate a hypothesis on how these variables influence the metabolism of Tetrahydrocannabinol (THC) and Cannabidiol (CBD).

**Results:**

Oral cannabis use was confirmed in 71 patients, with an average age of 68.5 years, and primarily white women. Of the 71 patients, 10 had no atypical variants; 31 had atypical variants in CYP2C9; 37 had atypical variants in CYP2C19; 6 had atypical variants in CYP3A4; and 15 had atypical variants in CYP3A5. Of the 71 patients, 5 were taking medications that could interact with THC, and 8 were taking medications that could interact with CBD.

**Conclusion:**

The results this study reveal the spectrum of hypothesized alterations in THC and CBD metabolism due to atypical genetic variants and medications. The absence of published clinical outcomes in this field renders it challenging to estimate clinical significance of these findings. Until such data become available, clinicians should remain aware of the possibility that atypical variants and medications may impact patients’ responses to THC and CBD.

## Introduction

Previous research has indicated that cannabinoid metabolism and patient responses can exhibit variability, even when comparable doses and formulations are administered (Bortolato et al. 2010; Qian et al. 2024). Factors influencing variability include high lipophilicity of cannabinoids, the impact of food on absorption, and tendency to accumulate in adipose tissue, and being highly protein bound (Chayasirisobhon 2020; Martinez Naya et al. 2024). Another potential explanation is genetic polymorphisms in genes responsible for cannabinoid metabolism (Wright 2024). For example, subsets of the population who have genetic variants or atypical phenotypes for CYP3A4, CYP2C9, and/or CYP2C19 genes may metabolize tetrahydrocannabinol (THC) and cannabidiol (CBD) differently than the general population (Table 1) (PHARMGKB n.d.). A third possibility is that interactions between cannabinoids and prescription medications via the CYP450 pathways (Bansal et al. 2023; Zendulka et al. 2016). Currently known CYP450 pathways influencing THC metabolism are CYP2C9, CYP3A4, and CYP3A5, and CBD metabolism are CYP2C19, CYP3A4, and CYP3A5 (EPIDIOLEX 2024; MARINOL. 2017). Given the increasing nationwide use of THC and CBD, knowledge of genetic polymorphisms and medication use that could potentially alter cannabinoid metabolism at least in a subset of patients may enable safer patient care (Wright 2024).

**Table 1:** Phenotype of genes encoding CYP450 enzymes, their function, and hypothesized effect on THC and CBD metabolism (PHARMGKB n.d.).

Phenotype	Function*	Hypothesized effect on THC/CBD metabolism
**Poor metabolizer**	Greatly reduced to no function of the CYP enzyme	Decreased metabolism of THC or CBD
**Intermediate metabolizer**	Moderately reduced function of the CYP enzyme	Decreased metabolism of THC or CBD
**Intermediate to normal metabolizer**	Mildly reduced function of the CYP enzyme	Decreased metabolism of THC or CBD
**Rapid metabolizer**	Moderately increased function of the CYP enzyme	Increased metabolism of THC or CBD
**Ultrarapid metabolizer**	Greatly increased function of the CYP enzyme	Increased metabolism of THC or CBD

*Abbreviations*: *CBD* cannabidiol, *THC* tetrahydrocannabinol

*Applies to CYP2C9, CYP2C19, and CYP3A4 but not CYP3A5. In the case of CYP3A5, intermediate and normal metabolizer phenotypes results in increased function and metabolism of the CYP enzyme

Our current understanding of the impact of atypical genetic variants and medications on THC and CBD metabolism is constrained by the scarcity of published literature. To date, only three published studies have reported the changes that atypical variants can have on THC and CBD levels or response (Davis et al. 2024; Sachse-Seeboth et al. 2009). The first study investigated the impact of the CYP2C9 *2 and *3 alleles, which are known to reduce CYP2C9 function (Sachse-Seeboth et al. 2009). In this study, oral THC was administered to 43 individuals, and the area under the curve (AUC) for THC was measured (Sachse-Seeboth et al. 2009). Individuals who were CYP2C9-poor metabolizers (*3/*3 genotype) exhibited a 70% increase in AUC for THC compared to normal metabolizers (Sachse-Seeboth et al. 2009). Additionally, CYP2C9-intermediate and -poor metabolizers (i.e., carriers of the *3 allele) showed a trend toward increased sedation with THC compared to normal metabolizers (Sachse-Seeboth et al. 2009). The second study evaluated the impact of CYP2C9 *2 and *3 alleles on THC metabolism, reporting higher THC levels in patients who were carriers of these alleles (Gasse et al. 2020). The third study associated genetic variations in CYP2C9 and CYP3A4 with negative effects of THC and cannabis use disorder, with sex-specific differences (Davis et al. 2024). However, research in this area is incomplete; the effects of variants within CYP2C19, and CYP3A5 genes that affect cannabinoid metabolism have not been described in clinical settings.

Similarly, there is only one in vivo study on the effect of medications on cannabinoid levels. In this phase I study, the impact of rifampicin (a strong inducer of CYP3A4) and ketoconazole (a strong inhibitor of CYP3A4) on the pharmacokinetics of THC and CBD was evaluated in healthy volunteers (Stott et al. 2013). The authors reported that CYP3A4 inhibitors and inducers significantly increased and decreased, respectively, the levels of both THC and CBD (Stott et al. 2013). Although there is limited clinical data at this point, we cannot ignore the possibility of varied THC or CBD metabolism in different patients due to atypical variants or use of medications than what is currently published.

In this context, this study aims to describe the presence of atypical pharmacogenomic profiles, the use of medications metabolized via CYP450 pathways in conjunction with THC and CBD, and to hypothesize their potential impact on THC and CBD metabolism in a nonselected sample of patients reporting oral cannabis use.

## Materials and methods

### Study design

We conducted a retrospective chart review on a cohort of unselected patients who had previously completed pharmacogenomic testing and reported cannabis use to their medical provider in a clinical encounter.

### Patients

For this study, we utilized data from the Mayo-Baylor RIGHT 10 K Study, which includes a cohort of over 10,000 patients who had previously undergone pharmacogenomic testing, specifically targeted oligonucleotide-capture sequencing of 77 pharmacogenes previously described (Wang et al. 2022). The results of these 77 pharmacogenes were integrated into the electronic medical records (EMR) to facilitate the incorporation of pharmacogenomic data into clinical practice and to provide practice-based alerts (Wang et al. 2022; Olson et al. 2013). Inclusion criteria included (1) research authorization, (2) adult (age 18 and older), and (3) documentation of oral cannabis use, as defined as “oral” or “by mouth” route of administration. Patients were excluded if cannabis use was only documented in clinical encounters that occurred outside of Mayo Clinic.

### Data collection

With electronic data search tools, we used the search terms CBD, THC, and cannabis to identify patients within this cohort who had oral cannabis use mentioned in their EMR. Clinical notes were manually reviewed to confirm use of cannabis and determine the date on which cannabis use was first documented in the EMR. Given the limited documentation on the exact composition of the cannabis products, we assumed all products had both CBD and THC. Demographics data, including age, sex, race, ethnicity, level of education, nicotine, and alcohol use were electronically and manually abstracted. Medication lists closest to the date of cannabis documentation were manually abstracted and pharmacogenomic phenotypes for CYP2C9, CYP2C19, CYP3A4, and CYP3A5 for each participant were electronically abstracted from their EMR.

### Study processes

Two pharmacists (JW and LH) with clinical expertise in pharmacogenomics reviewed medications in each participant to determine clinically relevant cannabis-drug interactions, as defined by involvement of strong or moderate inducers or inhibitors, that could potentially alter the metabolism of THC and CBD based on CYP450 metabolism pathways from UpToDate (n.d.). Based on the information provided in UpToDate, only strong or moderate inhibitors and inducers for CYP2C9, CYP2C19, CYP3A4, and CYP3A5 were deemed to be clinically relevant for the purposes of this study (UpToDate n.d.). Medications metabolized by CYP2C9, CYP3A4, and CYP3A5 were identified as potential THC-drug interactions. Similarly, medications metabolized by CYP2C19, CYP3A4, and CYP3A5 were identified as potential CBD-drug interactions. A list of strong and moderate inhibitors and inducers found in this sample are listed in column 2 of Tables 3 and 4. These phenotypes include intermediate to normal (*CYP2C9*, *CYP2C19*, *CYP3A4*, and *CYP3A5*), intermediate (*CYP2C9*, *CYP2C19*, *CYP3A4*, and *CYP3A5*), poor (*CYP2C9*, *CYP2C19*, and *CYP3A4*), rapid (*CYP2C19*), ultrarapid (*CYP2C19*), and normal (*CYP3A5*) metabolizers. Following this they hypothesized how the medications or atypical variants could influence the metabolism of THC and CBD, respectively, in each of the 71 patients.

## Results

A search for cannabis use within the RIGHT 10 K cohort (10,077 patients) on April 16, 2021, identified 164 individuals of which oral cannabis use was confirmed in 71 individuals, which was our sample. The average age of our sample was 68.5 years, was predominantly women (73.2%), of Caucasian race (94.4%), and non-Hispanic ethnicity (95.8%) (Table 2). On average, patients were on 8.1 medications not including vitamins or supplements. Of the 71 patients, 10 had no atypical variants; 31 had atypical variants in *CYP2C9*; 37 had atypical variants in *CYP2C19*; 6 had atypical variants in *CYP3A4*; and 15 had atypical variants in CYP3A5 (Table 3). The phenotype and genotype for each gene is listed in Table 3. Of the 71 patients, 5 were taking medications that could interact with THC, and 8 were taking medications that could interact with CBD.

**Table 2:** Demographic and clinical characteristics (*N* = 71)

Variable	No. (%)
Gender	
Male	19 (26.8)
Female	52 (73.2)
Age of cannabis documentation (average)	68.5
Race	
Caucasian	67 (94.4)
African American	1 (1.4)
Other	3 (4.2)
Ethnicity	
Hispanic	2 (2.8)
Non-Hispanic	68 (95.8)
Unknown	1 (1.4)
Alcohol use	
Current	49 (69.0)
Former	7 (9.9)
Never	15 (21.1)
Smoking status	
Current	7 (9.9)
Former	29 (40.8)
Never	35 (49.3)
Education level	
High school or less	6 (8.5)
Some college	25 (35.2)
Bachelor’s degree	16 (22.5)
Advanced degree	22 (31.0)
No information	2 (2.8)

**Table 3:** Distribution of CYP450 metabolism phenotypes in the sample

Gene, Phenotype, and genotype	Number
**CYP2C9 **	
** Poor metabolizer activity score 0 **	0
** Poor metabolizer activity score 0.5 **	1
*2/*3	1
** Intermediate metabolizer activity score 1.0 **	12
*1/*3	12
** Intermediate metabolizer activity score 1.5 **	18
*1/*2	18
** Normal metabolizer **	40
*1/*1	40
**CYP2C19**	
** Poor metabolizer **	1
*2/*2	1
** Intermediate metabolizer **	15
*1/*2	15
** Intermediate to normal metabolizer **	7
*2/*17	7
** Normal metabolizer **	34
*1/*1	34
** Rapid metabolizer **	12
*1/*17	12
** Ultrarapid metabolizer **	2
*17/*17	2
**CYP3A4 **	
** Intermediate to normal metabolizer **	6
*1/*22	5
*1/novel allele with heterozygous c.200A>G, p.Lys67Arg	1
** Normal metabolizer **	65
*1/*1	65
**CYP3A5**	
** Poor metabolizer **	56
*3/*3	55
*3/*3 with a heterozygous c.1111A>G, p.Ile371Val	1
** Intermediate metabolizer **	15
*1/*3	15

### Hypothesized influence on THC metabolism

Among the 71 patients, 4 had potential for both medication and gene interactions, 38 had potential for interactions involving single or combination genes only, 1 had potential for medication only interactions, and 28 had no relevant interactions with THC (Table 4). Information on inducers and inhibitors, atypical variants, and the hypothesized impact on THC metabolism for each patient is in Table 4.

**Table 4:** Hypothesized influence on THC metabolism

Participant	Medications: Inhibitors/Inducers	Atypical variants	Hypothesized influence on THC metabolism
1	Fluconazole strong inhibition of CYP3A4 and weak inhibition of CYP2C9	CYP2C9 IM	In this patient, two CYP pathways for THC metabolism could be downregulated by fluconazole. This patient, due to their CYP2C9 IM phenotype, has a genetic predisposition to reduced THC metabolism.
2,3	Diltiazem moderate inhibition of CYP3A4	CYP3A5 IM	In these two patients, one of the CYP pathways for THC metabolism could be downregulated by diltiazem. These two patients, due to their CYP3A5 IM phenotype, have a genetic predisposition to increased THC metabolism.
4	Carbamazepine strong CYP3A4 induction	CYP2C9 IM-NM	In this patient, one of the CYP pathways for THC metabolism could be upregulated by carbamazepine. This patient, due to their CYP2C9 IM-NM phenotype, has a genetic predisposition to reduced THC metabolism.
5		CYP2C9 PM	This patient, due to their CYP2C9 PM phenotype, has a genetic predisposition to reduced THC metabolism.
6–15		CYP2C9 IM	These patients, due to their CYP2C9 IM phenotype, have a genetic predisposition to reduced THC metabolism.
16–19		CYP3A4 IM-NM	These patients, due to their CYP3A4 IM-NM phenotype, have a genetic predisposition to reduced THC metabolism.
20		CYP2C9 IM-NM, CYP3A4 IM-NM, and CYP3A5 IM	This patient, due to their complex gene profile (CYP2C9 IM-NM, CYP3A4 IM-NM, and CYP3A5 IM), may have a genetic predisposition to altered THC metabolism. The net genetic impact on the metabolism is currently unknown.
21		CYP2C9 IM and CYP3A5 IM	This patient, due to their complex gene profile (CYP2C9 IM, and CYP3A5 IM), may have a genetic predisposition to altered THC metabolism. The net genetic impact on the metabolism is currently unknown.
22–34		CYP2C9 IM-NM	These patients, due to their CYP2C9 IM-NM phenotype, have a genetic predisposition to reduced THC metabolism.
35		CYP2C9 IM-NM and CYP3A4 IM-NM	This patient, due to their CYP2C9 IM-NM and CYP3A4 IM-NM phenotype, has a genetic predisposition to reduced THC metabolism.
36–42		CYP3A5 IM	These patients, due to their CYP3A5 IM phenotype, have a genetic predisposition to increased THC metabolism.
43	Diltiazem moderate inhibition of CYP3A4		In this patient, one of the CYP pathways for THC metabolism could be downregulated by diltiazem.
44–71	N/A		These patients had no medication or gene interactions that could alter THC metabolism.

*Abbreviations:* *IM* intermediate metabolizer, *IM-NM* intermediate-to-normal metabolizer, *N/A* not applicable, *PM* poor metabolizer, *RM* rapid metabolizer, *THC* tetrahydrocannabinol, *UM* ultrarapid metabolizer

### Hypothesized influence on CBD metabolism

Among the 71 patients, 4 had potential for both medication and gene interactions, 36 had potential for interactions involving single or combination genes only, 4 had potential for medication only interactions, and 27 had no relevant interactions with CBD (Table 5). Information on inducers and inhibitors, atypical variants, and their hypothesized impact on CBD metabolism for each patient is in Table 5.

**Table 5:** Hypothesized influence on CBD metabolism

Participant	Medications: Inhibitors/Inducers	Atypical variants	Hypothesized influence on CBD metabolism
1	Fluoxetine moderate CYP2C19 inhibition	CYP2C19 PM	In this patient, one CYP pathway for CBD metabolism could be downregulated by fluoxetine. This patient, due to their CYP2C19 PM phenotype, has a genetic predisposition to reduced CBD metabolism.
2	Diltiazem moderate inhibition of CYP3A4	CYP3A5 IM	In this patient, one CYP pathway for CBD metabolism could be downregulated by diltiazem. This patient, due to their CYP3A5 IM phenotype, has a genetic predisposition to increased CBD metabolism.
3	Fluoxetine moderate inhibition of CYP2C19	CYP3A5 IM	In this patient, one CYP pathway for CBD metabolism could be downregulated by fluoxetine. This patient, due to their CYP3A5 IM phenotype, has a genetic predisposition to increased CBD metabolism.
4	Diltiazem moderate inhibition of CYP3A4	CYP2C19 RM CYP3A5 IM	In this patient, one CYP pathway for CBD metabolism could be downregulated by diltiazem. This patient, due to their CYP2C19 RM and CYP3A5 IM phenotype, has a genetic predisposition to increased CBD metabolism.
5,6		CYP2C19 UM	These patients, due to their CYP2C19 UM phenotype, have a genetic predisposition to increased CBD metabolism.
7–9		CYP2C19 RM and CYP3A5 IM	These patients, due to their CYP2C19 RM and CYP3A5 IM phenotype, have a genetic predisposition to increased CBD metabolism.
10–16		CYP2C19 RM	These patients, due to their CYP2C19 RM phenotype, have a genetic predisposition to increased CBD metabolism.
17–20		CYP3A5 IM	These patients, due to their CYP3A5 IM phenotype, have a genetic predisposition to increased CBD metabolism.
21–32		CYP2C19 IM	These patients, due to their CYP2C19 IM phenotype, have a genetic predisposition to reduced CBD metabolism.
33		CYP2C19 IM and CYP3A4 IM-NM	This patient, due to their CYP2C19 IM and CYP3A4 IM-NM phenotype, has a genetic predisposition to reduced CBD metabolism.
34,35		CYP2C19 IM-NM	These patients, due to their CYP2C19 IM-NM phenotype, have a genetic predisposition to reduced CBD metabolism.
36,37		CYP2C19 IM-NM and CYP3A4 IM-NM	These patients, due to their CYP2C19 IM and CYP3A4 IM-NM phenotype, have a genetic predisposition to reduced CBD metabolism.
38,39		CYP3A4 IM-NM	These patients, due to their CYP3A4 IM-NM phenotype, have a genetic predisposition to reduced CBD metabolism.
40	Carbamazepine strong induction of CYP3A4		In this patient, one CYP pathway for CBD metabolism could be upregulated by carbamazepine.
41	Fluconazole strong inhibition of CYP2C19 and CYP3A4		In this patient, one CYP pathway for CBD metabolism could be downregulated by fluconazole.
42	Diltiazem moderate inhibition of CYP3A4		In this patient, one CYP pathway for CBD metabolism could be downregulated by diltiazem.
43	Fluoxetine moderate inhibition of CYP2C19		In this patient, one CYP pathway for CBD metabolism could be downregulated by fluoxetine.
44–71	N/A		These patients had no medication or gene interactions that could alter CBD metabolism.

*Abbreviations:* *CBD* cannabidiol, *IM* intermediate metabolizer, *IM-NM* intermediate to normal metabolizer, *N/A* not applicable, *PM* poor metabolizer, *RM* rapid metabolizer, *UM* ultrarapid metabolizer

## Discussion

The results of our study highlight the spectrum of potential alterations in THC and CBD metabolism that may arise due to atypical genetic variants and medications and is the first study to report this range of possibilities. Given the limited literature, assessing the clinical significance of these interactions is challenging. However, until more evidence emerges, clinicians should remain aware of their potential impact on patients’ responses to THC and CBD.

From a pharmacogenomic perspective, although atypical variants may influence cannabinoid metabolism, the greatest clinical value may reside in identifying patients with extreme phenotypes. These individuals potentially exhibit a higher likelihood of altering THC or CBD metabolism, which may impact therapeutic outcomes. This includes poor metabolizers of CYP2C9, CYP2C19, or CYP3A4, normal metabolizers of CYP3A5, and ultrarapid metabolizers of CYP2C19. In our sample that was predominantly Caucasian, only three of the 71 patients were poor metabolizers for CYP2C9 (1.4%) or CYP2C19 (2.8%), consistent with the prevalence of CYP2C9 and CYP2C19 poor metabolizers within Caucasian populations (1% and 2%-5%, respectively) (Belle and Singh 2008). Our sample did not contain any patients with extreme CYP3A4 nor CYP3A5 phenotypes, which is not surprising because it is rare in the Caucasian population. This suggests that only a small percentage of patients in a Caucasian sample may experience notable baseline alterations in THC or CBD metabolism. Although we observed several other atypical variants (*n*= 42), we believe that over 90% of these are unlikely to have clinical significance. The overall distribution of atypical variants in our sample is consistent with previous studies, which report atypical variants for CYP2C9 and CYP2C19 phenotypes ranging from 37 to 39% and 54–60%, respectively, in populations of Caucasian ancestry (Wright 2024; PHARMGKB n.d.; Bielinski et al. 2020). However, these results cannot be generalized to non-Caucasian samples, and future prospective studies evaluating whether THC and CBD metabolism varies in diverse populations may be of value.

The results of our study suggest that CYP pathways for THC and CBD metabolism may be influenced by medications. This may be particularly relevant for medications that are moderate to strong inhibitors or inducers of CYP2C9, CYP2C19, and CYP3A4, as they can have a higher likelihood to alter cannabis metabolism. According to the FDA, moderate inhibitors increase the AUC by 2-fold up to 5-fold, while strong inhibitors increase the AUC by more than 5-fold (FDA. 2023). In this context, relevant inhibitors and inducers in our sample included fluconazole, diltiazem, fluoxetine (inhibitors), and carbamazepine (inducer). While these medications were specific to our sample, other medications could also potentially influence cannabinoid metabolism. Currently, there is no comprehensive resource or tool that lists all these medications. However, commonly prescribed medications that may be relevant in this context include moderate to strong inhibitors of CYP2C9, CYP2C19, and CYP3A4 such as omeprazole, azithromycin, verapamil, fluvoxamine, sulfamethoxazole/trimethoprim, and nirmatrelvir-ritonavir. A detailed list of these medications is beyond the scope of this paper.

Based on the current data, it is premature to recommend pharmacogenomic testing for all patients who use cannabis. Since pharmacogenomic testing has become more common, it may be beneficial to ask patients if they have undergone such testing and to incorporate these results into their medication evaluations. For patients who have not had pharmacogenomic testing, it may be worth considering if they experience unexpected serious adverse effects. As a medical community, we are still working to understand the complexities of appropriate cannabis use. Currently, there is a lack of clinical guidelines to predict adverse effects of cannabis resulting from its metabolism. Until such information is available, awareness of atypical cannabinoid metabolism resulting from atypical variants and medications, and consultation with a clinician such as a pharmacist with expertise can help guide clinical care.

### Strengths and limitations

One of the strengths of our study is that it is the first to report atypical variants of CYP2C9, CYP2C19, CYP3A4, and CYP3A5 phenotypes identified through sequencing in a clinical sample of cannabis users. Sequencing can detect rare variants that genotyping might miss, providing a more comprehensive view. Previous studies that used genotyping to examine CYP2C9 and THC interactions only evaluated CYP2C9 *2 and *3 variants and could have missed important alleles that resulted in false-normal interpretation of the CYP2C9 phenotype (Gasse et al. 2020; Sachse-Seeboth et al. 2009). In contrast, our study that interrogated all variants in the CYP2C9 gene reassured us that the CYP2C9 phenotype interpretation was comprehensive. Another strength of our study is the concurrent reporting of both atypical variants and medications that influence cannabis metabolism in a clinical sample.

Our study had several limitations. First, we used a convenience sample that required documentation of cannabis use in EMR, which might have led to missing patients who used cannabis but did not report it, or providers who did not document cannabis use reported by patients, and self-reporting bias. Second, details related to cannabis use, such as dosing, formulation, and frequency, were often poorly documented, leading us to assume all documented cannabis contained both THC and CBD, which might not have been accurate. Third, determining if patients were using cannabis and interacting medications simultaneously from retrospective chart reviews was challenging. We assumed continuous cannabis use for our hypothesis, but intermittent use might not produce as prominent of an effect in terms of cannabis accumulation. These limitations highlight the current challenge of how cannabis is documented in medical records. Fourth, there is a complex bidirectional relationship between cannabis and medications. Since our primary aim was to evaluate the effect of medications on cannabinoid metabolism, we intentionally focused on this aspect of the interaction. We acknowledge that cannabis can affect medications levels, however, this was not incorporated in the analysis of our study since this was not our primary aim. Fifth, we acknowledge there are other pathway for cannabinoid-drug-gene interactions such as uridine glucuronosyltransferase (UGT) enzymes. However, the pharmacogenomic testing that was done as part of the RIGHT10K did not report UGT genes in the electronic medical records. Therefore, no information regarding UGT enzymes was available for this sample. We acknowledge that this may be a limitation. Sixth, patients with certain ancestries, particularly South Asian, may have a higher risk of atypical phenotypes affecting THC and CBD metabolism (Zhou and Lauschke 2022). Since our convenience sample was predominantly Caucasian, our results may not be generalizable to other ancestries. Finally, our sample included mostly older patients. It is known that the expression and activity of CYP450 enzymes vary with age, which can influence medications metabolism and should be considered (Corton et al. 2022; Tanaka 1998). Despite these limitations, our study adds important information to the current scant body of literature and may be beneficial as a hypothesis to support future clinical studies evaluating these questions.

## Data Availability

No datasets were generated or analysed during the current study.
